# Micro-level explanations for emergent patterns of self-governance arrangements in small-scale fisheries—A modeling approach

**DOI:** 10.1371/journal.pone.0175532

**Published:** 2017-04-13

**Authors:** Emilie Lindkvist, Xavier Basurto, Maja Schlüter

**Affiliations:** 1 Stockholm Resilience Centre, Stockholm University, Stockholm, Sweden; 2 Duke University Marine Lab, Nicholas School of the Environment, Duke University, Beaufort, NC, United States of America; University of Waterloo, CANADA

## Abstract

Small-scale fisheries (SSFs) in developing countries are expected to play a significant role in poverty alleviation and enhancing food security in the decades to come. To realize this expectation, a better understanding of their informal self-governance arrangements is critical for developing policies that can improve fishers’ livelihoods and lead to sustainable ecosystem stewardship. The goal of this paper is to develop a more nuanced understanding of micro-level factors—such as fishers’ characteristics and behavior—to explain observed differences in self-governance arrangements in Northwest Mexico. We focus on two ubiquitous forms of self-governance: hierarchical non-cooperative arrangements between fishers and fishbuyers, such as patron-client relationships (PCs), versus more cooperative arrangements amongst fishers, such as fishing cooperatives (co-ops). We developed an agent-based model of an archetypical SSF that captures key hypotheses from in-depth fieldwork in Northwest Mexico of fishers’ day-to-day fishing and trading. Results from our model indicate that high diversity in fishers’ reliability, and low initial trust between co-op members, makes co-ops’ establishment difficult. PCs cope better with this kind of diversity because, in contrast to co-ops, they have more flexibility in choosing whom to work with. However, once co-ops establish, they cope better with seasonal variability in fish abundance and provide long-term security for the fishers. We argue that existing levels of trust and diversity among fishers matter for different self-governance arrangements to establish and persist, and should therefore be taken into account when developing better, targeted policies for improved SSFs governance.

## Introduction

Small-scale fisheries (SSF) in developing countries are expected to play a significant role in reducing poverty, providing sustainable livelihoods, and improving ecosystem stewardship in the decades to come [1,2]. Whether this potential is realized strongly depends on how they are governed. Many SSFs around the world are self-governed through informal arrangements [3,4] or co-managed through collaboration between fishers, managers and scientists [5]. Self-governance arrangements come in many different forms, which can be classified along a continuum with two opposing positions: hierarchical non-cooperative structures between fishers and fishbuyers, such as patron-client relationships (PCs), versus cooperative structures amongst fishers, such as fishing cooperatives (co-ops) [6]. Both structures have documented benefits and drawbacks for human well-being and ecosystem stewardship [6–12]. While the understanding of factors that enable successful self-governance in SSFs around the world is well-developed [6,9,13,14] explanations for the diversity of self-governance arrangements and their implication for sustainable management are scarce [6]. The goal of this paper is to enhance understanding of the daily interactions between fishers, fishbuyers and the fish stocks that shape these informal arrangements and shed light on the conditions under which different forms of self-governance may become established and persist.

We stipulate self-governance arrangements as “[…] those where the individuals involved in fishing activities take on the duties of designing agreements related to fish production and commercialization, and monitor and enforce their commitments to each other without resorting to the state or other external authorities” ([6] p.2). The success of these arrangements strongly depends on their capacity to deal with free-riding and non-compliance of their members [13]. Extensive empirical research has identified trust and social norms as important factors in ensuring compliance and collective action for PCs and co-ops alike [9,10,13–16]. The level of trust or existence of social norms in a community, can therefore not explain observed differences in the distribution of PCs and co-ops within similar contextual settings (i.e. degree of government support for co-ops, cultural settings, political settings, etc.) alone. Here the micro-level dynamics of trust may become important, i.e. how trust evolves and erodes between individuals in their daily fishing and trading activities may shape the success or failure of different self-governance forms within a given context [17].

In SSFs, fishers do not only interact with other fishers or fishbuyers to organize their fishing operations but also with the ecosystem, i.e. the fish stocks. In doing so, an SSF can be perceived as a so-called social-ecological system [18]. In social-ecological systems, institutions (i.e. the rules in use; [19]) and human behavior are shaped by social and biophysical factors and processes alike [20,21]. Changes in biophysical factors such as increased variability in resource availability caused by the effects of global environmental change can thus potentially have significant effects on the dynamics of SES [22,23]. Just as social-ecological systems, SSFs are complex adaptive systems [24] where interactions between individuals at the micro-level affects the community (macro-level), which then resonates back and affects the behavior of the same individuals. The processes how micro-level behaviors and interactions form trust, and how they interact with other social and ecological variables, to shape the emergence of a prevailing self-governance arrangement at the macro-level is poorly understood [25].

Following the seminal paper of Sethi and Somanathan, [26], a suite of studies have investigated social norms as a mechanism for cooperation in common pool resource dilemmas, focusing on the effect of network structures [27,28], ostracism [29], social exclusion [30] or the robustness of norm-driven cooperation [31]. These models are purely theoretical and apply an evolutionary game theoretic approach to study the evolution of cooperation in a homogenous human population given certain disapproval or punishment mechanisms. Understanding how micro-level behaviors of heterogeneous actors and their interactions with other actors and, their biophysical environments, give rise to different forms of self-governance, however, necessitates a disaggregated approach. Agent-based modeling is a computational approach that allows incorporating diversity among actors and their interactions to study emergent group or community level outcomes [21,25,32–34]. In the realm of social-ecological systems, and similar to the game theoretic models mentioned above, most agent-based models focus on cooperation among resource users for sustainable resource management [32], and some focus more specifically on the evolution of institutions for sustainable resource management [35–37]. Our study extends this body of research by going beyond cooperation around extraction of the resource to include; cooperation for the organization of fishing activities and the dynamics of trust necessary for cooperation on a daily basis, for example in the exchange of loans and the selling of the catch [38].

In this paper we thus address significant research gaps by explicitly focusing on the micro-level mechanisms, particularly those mechanisms related to different dimensions of trust in fishers’ daily fishing and trading operations, dimensions that might well explain both the establishment, and the persistence of PCs and co-ops. We investigate the interplay between the individuals (micro-level), the organizations (meso-level) and the fishing community (macro-level), and the fish resource they depend upon. To this end, we have developed an agent-based model that formalizes key hypotheses of SSFs in Northwest Mexico, building on qualitative interview data, long-term observations, logbook data and literature. The model serves as a virtual laboratory to operationalize the following research questions; a) What is the effect of micro-level factors related to trust—such as fishers’ reliability, and loyalty between fishbuyers and fishers and between members in co-ops—for the establishment and persistence of different self-governance arrangements? b) How does environmental variability affect whether co-ops or PCs will emerge as the dominant form of self-governance? c) How stable are these two self-governance arrangements and what makes them fail? We argue that this in-depth understanding of micro-level mechanisms and the interplay between micro-, meso- and macro-institutional levels is a fundamental step towards developing better, targeted policies for improving fishers’ livelihoods and marine ecosystem stewardship globally.

The paper proceeds as follows. First we present a background on cooperative and non-cooperative self-governance in SSF, and introduce our example case study. Second, we introduce the empirical base for our choice of explanatory variables, and link the empirical base to our agent-based model and the simulation experiments that we use to answer our research questions. Third, we present the results, and end with a discussion of our findings and some concluding remarks.

### Cooperative and non-cooperative self-governance of small-scale fisheries in Northwest Mexico

SSFs in Northwest Mexico play an important social and economic role at regional and national levels [39,40]. They are often self-governed by co-ops which have been promoted by the Mexican government as a way to strengthen the social and economic development of the sector since the 1930s [41–43]. Constitutional reforms in 1992 expanded some of the state benefits previously reserved for co-ops (e.g., fishing permits, loans and subsidies) to private corporations and individuals [41–44]. Since then, PC forms of self-governance, which were legalized in 1947, seem to be rapidly on the rise. It is estimated that 30% of the fishers are now working under PC arrangements [17].

Fisheries in Mexico have to cope with highly variable environmental conditions [45]. Seasonal variability caused by wind conditions in the southern Gulf of California or El Niño events affect both stock abundance and catch rates [45,46]. As shown elsewhere, PCs are an institutional adaptation to environmental constraints and uncertainties as they provide insurance to fishers during periods when catches are low [9]. At the same time, the institution of PCs itself have shown high adaptive capacity to social and environmental change [9]. Co-ops can under certain circumstances perform equally well when subject to environmental fluctuations [40,44,46,47]. In Northwest Mexico, for instance, co-ops have successfully adapted to El Niño events by developing adaptive rules and practices [48]. For other co-ops, diversifying their livelihood activities to include tourism helps them cope with low catches [49]. Hence, the interplay between fish resource dynamics, environmental variability and social processes is important for the emergence and maintenance of self-governance in SSF.

PCs and co-ops also face challenges related to compliance and free riding, as the self-interests of the fisher and its co-op or patron do not necessarily align. PCs are often based on informal contracts between a fishbuyer and a fisher. Fishers contract with fishbuyers because patrons own the capital means of production or are in control of the commercialization channels. Thus, fishbuyers can lend daily capital to the fisher to buy fishing supplies (e.g., bait, gasoline, etc.) against the expectation that the catch will be brought back to the fishbuyer [9,50,51]. As in any economic exchange with information asymmetries, PCs often face principal-agent problems (e.g. [52,53]) when the self-interests of the principal (fishbuyer) and agent (fisher) do not overlap. A fisher who seeks to maximize his individual economic gains may thus face incentives to deceive the fishbuyer on the repayment of the loan or the landing of the catch. It is therefore in the interest of the fishbuyer to seek out and contract with reliable fishers to minimize the possibility of economic loss [38]. Observations in Northwest Mexico show that fishbuyers often terminate their relationship with a fisher after only a few transactions if they are not satisfied with the reliability of the fisher [17,38,43,54]. According to one fishbuyer in Northwest Mexico, on average, 20% of the fishers he lends capital to do not return with catch [38]. The issue of breaking an agreement or not fulfilling the agreed upon tasks (which in the following we will term “cheating”) has been raised in interviews with fishbuyers and co-op leaders in different parts of Mexico [6,7,38,55]. In the community of Kino Bay in Northwest Mexico, fishbuyers reported having a small group of highly loyal fishers with whom they interact and a large fraction of fishers they only interact with occasionally, presumably because they are less trustworthy [38].

In contrast, co-ops are often built on formal contracts that regulate membership to the association, the collective ownership of fishing property-rights, equipment and financial resources, the organization of fishing and commercialization activities, and the allocation of benefits resulting from fishing, including social security and healthcare [7,55,56]. Co-ops often face collective action problems given that members can shirk their responsibilities and still enjoy the benefits collectively produced. Similar to PCs, fishers face incentives to cheat by landing their catch elsewhere if the benefits outweigh the costs [13,25]. Additionally, co-ops need to overcome high transaction costs during their establishment, including e.g. the costs of reaching joint agreements [13]. These costs can be a significant barrier for successful cooperation. However, homogeneity within groups (e.g. shared skills such as diving skills or social equity), small-group size, kinship or common social history can reduce these transaction costs [57]. In Northwest Mexico, co-ops’ success has been associated with a number of factors, including having a history of working together or a sense of community, which helps to develop norms of fairness and transparency, accountability and high quality knowledge and decision-making [6,7].

Social norms based on trust and reciprocity have been identified as important factors to overcome the principal-agent or collective action problems inherent in any form of self-governance including those considered in this study [13]. Crona et al. [10], for instance, showed that fishbuyers in East Africa regularly issue loans based on trust and that fishers are bound to a fishbuyer through strong social norms and a mutual agreement to sell their fish to the dealer they work with. Similarly, Johnson highlights the importance of reciprocal relationships for PCs in India [9]. Empirical studies on co-ops and PCs in Northwest Mexico have highlighted the importance of the reliability of fishers. Furthermore, trust and reciprocity for the establishment and maintenance of successful interactions between a fisher and a fishbuyer or between fishers within a co-op are both shown to be important [6,7,17,38,42]. Contrary to the studies from other countries, however, social norms do not seem to be sufficiently developed in Northwest Mexico to prevent cheating in all but a few cases. Micro-level factors, such as the different dimensions of trust, and their interactions with the social-ecological environment that may give rise to high loyalty or a community social norm and thus enable cooperative or non-cooperative self-governance, have however been less studied. With this study we aim to address this gap.

## The small-scale fishery model

**Fig 1:**
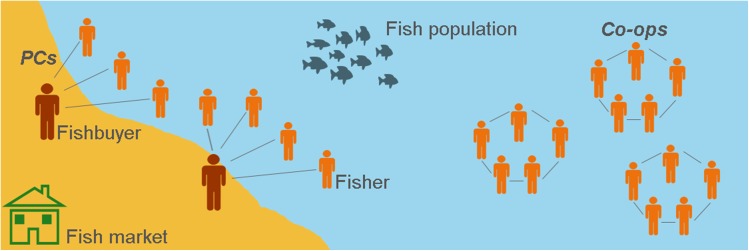
The environment of the fishery model. **The fishery model consists of fishers, fishbuyers, a fish population, and a fish market.** Fishers can be organized in either a PC by a link to a fishbuyer, or in a co-op by links to other fishers (members).

The purpose of the agent-based SSF model SMILI (**Sm**all-scale fisheries: **I**nstitutions and **L**ocal **I**nteractions; [58]) is to explain the establishment and persistence of co-ops and PCs by exploring the interactions of selected micro-level attributes and behaviors of fishers and fishbuyers within a dynamic social and ecological environment. The model consists of fishers and fishbuyers (‘actors’), the fish population and the fish market (‘entities’), and co-ops and PCs (social networks; see Fig 1 for a conceptual model of the fishery). A PC is defined as a network of fishers with the fishbuyer as the central hub. The fishbuyer is linked to fishers through the loyalty between them. A co-op is defined as a fully interconnected network structure among all members (Fig 1). Fishers, fishbuyers and co-ops are characterized by attributes that affect their fishing and selling behavior.

**Fig 2:**
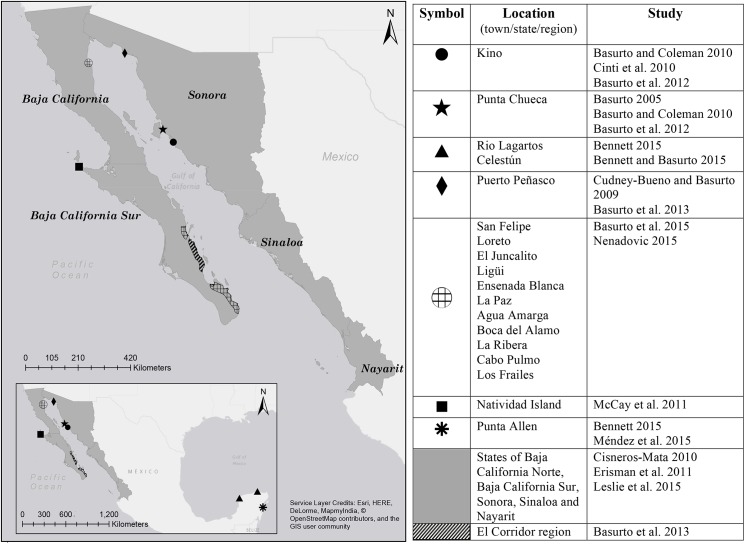
Map of sites and studies that have informed our understanding of self-governance in small-scale fisheries in Mexico. The base map was obtained from http://www.esri.com/data/basemaps.

Model design (i.e. selection of variables as well as key interactions), model analysis (i.e. design of simulation experiments), and model validation were based on empirical hypotheses about variables that may explain the establishment and persistence of the two forms of self-governance, data, expert knowledge, and theory. Primary and secondary data are largely from Northwest Mexico, however, we also included studies from other areas in Mexico when appropriate (e.g. to inform the modeling of fisher interactions as members of a co-op; Fig 2). Moreover, one of the authors has spent more than 15 years studying small-scale fisheries in Northwest Mexico, including many in-depth field studies. This includes participant observations in a number of fishing communities in Northwest Mexico since 1998 when the co-author lived in the community of Kino Bay for a year. After that, annual visits have been conducted and long-term relationships forged with a number of fishers and fishbuyers in the region. We build on this knowledge and experience when there is a lack of data. The development of the model followed an iterative approach where gaps in understanding were addressed by going back to the field to conduct follow-up interviews with fishers and fishbuyers in a multi-year process of alternating field research and model development, the results of which are presented in Basurto [38]. Note that our approach differs from a multiple N case study design as it uses an agent-based model built on a synthesis of empirical knowledge to explore hypotheses about possible causal mechanisms that explain empirical observations such as the diversity of governance forms [34]. We do so by using the model to identify which configurations of individuals and interactions give rise to the different self-governance arrangements.

In the following section, we first describe the empirical motivation for the selection of the variables and processes represented in the model and their formalization. Second, we describe the activities implemented in the model and the corresponding empirical motivation. Finally, we provide the design of the simulation experiments. Model assumptions, their empirical justification and model interpretation are summarized in S1 Table. To accommodate readers less familiar with agent-based modeling terminology, we use terminology in line with a fishery, rather than modeling terms, whenever possible.

### Variable selection—Empirical motivation

As a first step of identifying key variables that may explain the diversity of governance forms we used the SES framework of Ostrom [20]. The framework application provided guidance for building hypotheses about the causal effects on collective action of selected resource system variables such as the predictability of the resource, governance system variables such as the property rights system (licensing), and attributes of the user group such as social norms or past experiences. Based on literature, empirical studies and experience with PCs and co-ops from the region we focused on social norms, past experiences and predictability of the resource as possible explanatory factors. Property rights and cultural context do not vary at our level of analysis, which is the community. In order to understand the coexistence or dominance of one governance arrangement over the other in communities that are located in similar socio-political and biophysical settings we hypothesized that we had to go beyond studying social norms to understand the dynamics of trust that underlies successful interactions between a fisher and a buyer or members of a co-op. Empirical research has revealed a set of important micro-level behavioral variables, such as the social class of a buyer or his/her social skills [38]. As a starting point, and to reduce complexity, we however, decided to focus on different dimensions of trust which were considered most relevant for explaining the establishment and persistence of both forms [59]. Based on primary and secondary data as well as expert knowledge on small-scale fisheries interactions in Northwest Mexico, we operationalized trust as *fisher reliability*, *loyalty*, and *history of working together*. We briefly explain the three dimensions and their empirical basis below and in Table 1.

**Table 1:** Main attributes of the fishers, fishbuyers and co-ops, their empirical definitions and model interpretation.

Attribute	Empirical Definition	Model interpretation
**Fisher**		
Reliability	The trustworthiness of a fisher that he will ‘do what he said he would do’.	A fixed attribute of a fisher randomly drawn from a normal distribution with mean m and standard deviation sd.
Loyalty	Reciprocated trust from a fisher to his buyer or co-op.	A dynamic attribute of a fisher with an initial value of 0 for a fisher working with a fishbuyer and an initial value set to “initial loyalty” for a fisher working in a co-op.
Initial loyalty	Established loyalty resulting from a history of working together.	The initial value of loyalty (“initial loyalty”) of a fisher working in a co-op.
Fishing skills	A fisher’s skill to deliver the agreed catch. E.g. knowing where to find the fish, and catching the right target species.	A fixed parameter randomly drawn from a normal distribution with mean m and standard deviation sd.
Reputation	A combination of a fishers trustworthiness and fishing skills	Reliability × fishingskills
Capital	Financial assets of fishers.	A dynamic attribute of a fisher
**Buyer**		
Capital	Financial assets of fishbuyers	A dynamic attribute of a fishbuyer.
Loyalty growth rate	The rate at which loyalty builds up between fishers and their fishbuyer.	A fixed parameter that determines the speed of loyalty increase between a fishbuyer and a fisher in a PC.
**Co-op**		
Capital	The co-op’s shared assets	A dynamic attribute of a group of co-op fishers that is calculated as the sum of all members’ capital.
Co-op loyalty	The accumulated trust between the co-op members	A dynamic variable that is calculated as the sum of all members’ loyalty
Loyalty growth rate	Co-ops face higher transaction costs when starting up their business, which makes it difficult to build up loyalty compared to PCs.	A fixed parameter that determines the speed of loyalty increase between a co-op and a fisher. It is set to 50% of the loyalty growth rate of PCs.

Loyalty and reliability can be interpreted as two concepts closely related to trust. According to Glaeser [60], trust is defined as the willingness to take a risk based on positive expectations of future reciprocity from the trustee. We define loyalty as a form of accumulated or reciprocated trust, and reliability as a form of trustworthiness closely linked to ones’ reputation as a fisher and member of a fishing community. Reliable fishers are those fishers who “do what they said they would do,” who maintained their fishing rigs in good working condition, showed up ready to go fishing when the weather was good for fishing, and had good *fishing skills* (i.e., provide the agreed quantity and species). However, fishbuyers often state that they need to make a trade-off between fishers with high reliability versus excellent fishing skills. Fishers that score high on both these characteristics are very hard to come by [38]. A history of working together, i.e. previous successful interactions between the same fishers, can help to overcome the high transaction costs a co-op faces during the establishment phase (e.g. calling members for meetings, establishing entry, exit and working rules for the co-op), where previous cooperation lowers these transaction costs significantly [6,55].

### Interpretation of actor attributes in the model

The variables loyalty, reliability and history of working together were formalized as attributes of each fisher agent. Reliability was interpreted as a fixed personality trait, while loyalty is a dynamic variable, which increases or decreases with each fisher–fishbuyer or fisher–co-op interaction according to a fixed loyalty growth rate. The initial value of loyalty is interpreted in the model as a proxy for the co-op members’ history of working together.

Fishers also have fixed fishing skills and a reputation, which is a combination of their fishing skills and reliability. Fishbuyers know the reputation of a limited number of fishers in the community. Fishbuyers will aim to attract fishers who have a reputation of high reliability and are more likely to continue working with those that show loyalty [38]. Co-ops also aim to attract trustworthy and loyal fishers to their associations [55].

Fishers and fishbuyers have financial capital, which increases or decreases with each fishing and financial interaction. Each fishbuyer and co-op has an equal and fixed market share, which determines its fish demand. Additionally, the group of fishers working together in a co-op has the attributes *coop-capital* and *coop-loyalty*. Table 1 provides an overview of the empirical definitions of actors’ attributes and the respective implementation in the model. The calculations and parameter settings for the model can be found in S1 Calculations.

### Interpretation of activities and processes in the model

#### Daily fishing activities

**Fig 3:**
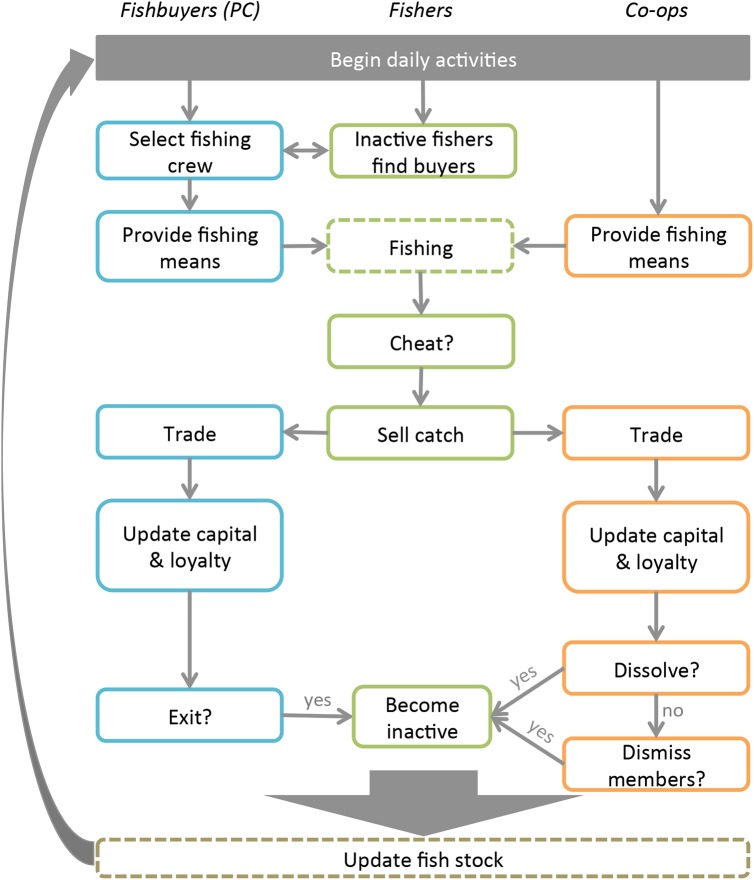
The daily activities in the fishery model. The fishbuyers, fishers and co-ops all take part in the daily activities. The activities start with fishbuyers informally contracting with fishers, then fishbuyers and co-ops provide means for fishing, and the activities continue as indicated by the arrows. By the end of each day the fish stock is updated with intrinsic growth rate and mortality, and the activities start over again the next day (time step).

The fishery consists of 100 fishers. Fishers that are organized in co-ops or PC relationships will go fishing, and are termed ‘active fishers’. Fishers that are not members of any PC or co-op are referred to as ‘inactive fishers’ and do not go fishing. Fishing pressure thus varies depending on the number of active fishers. Fig 3 provides an overview of a day in the fishery, i.e. the sequence of events from the preparations to go fishing to the assessment of the financial (capital) and social (loyalty) state of a PC and co-op at the end of the day. History of working together, loyalty, and reliability play central roles in the development of daily fishing activities because they influence all key aspects related to fishing and trading catch. To explain and justify our interpretation of the empirical data on the fishing process, we provide a step-wise description of these daily activities in Table 2, their empirical definition, and the model interpretation of these steps.

**Table 2:** The key steps of the daily fishing activities for the members of PCs and co-ops and how these daily processes are implemented in the model.

Step	Description based on empirical observations in Northwest Mexico	Model interpretation
1. Select fishing crew:	A fishbuyer and a fisher agree to work together. Fishers look for reliable buyers and buyers for reliable fishers Buyers will take on fishers based on market demand. Co-ops do not establish daily working contracts, because they have fixed members.	Based on reliability and fishing skills fishbuyer and fishers establish a working contract. If the buyer is under-supplied he will look for new fishers, but also fishers approach buyers. This process iterates between a buyer choosing the fisher with the highest reputation, and a buyer being approached by a random inactive fisher. This iteration continues until the buyer’s demand is fulfilled or when there are no more inactive fishers (S1 Calculations Eq 14 and Eq 15). Co-ops have fixed membership and thus do not select a fishing crew.
2. Provide fishing means:	Fishbuyer provides means of production to the fisher in the form of equipment (i.e., boat, fishing gear) and/or a loan for gas, bait, and/or food for the trip. Co-ops usually have this addressed within the co-op.	Fishers borrow money for their fishbuyer or co-op equivalent to the cost of going fishing. Fishbuyers and co-ops capital is reduced.
3. Fishing:	Fishers go fishing and returns with catch.	Fishers go fishing and return with catch, fish stock is updated.
4. Catch landing and selling (cheat?):	Catch is landed to the fishbuyer or co-op. However, if a better price is offered by another fishbuyer or co-op a fisher might cheat on their agreement and sell part of the catch elsewhere, or not deliver any catch. This depends on the reliability of the fisher and its loyalty to present organization.	We assume that there is always an incentive to cheat. Based on the fishers’ reliability and the loyalty formed to the fishbuyer or among member in the co-op, the fisher will decide to cheat and sell the catch to a different fishbuyer or co-op. Let *reliability* = fisher’s reliability, *loyindex* = fishers’ loyalty index calculated as Eq 22 in S1 Calculations, *α* and ***β*** be random numbers between [0…1] a fisher will cheat if: *reliability > α AND loyindex< β*
5. Trade:	Catch is sold and fishbuyer makes profit. In co-ops profit from trading catch can be allocated in a diversity of ways between members, e.g. distribution of fishers’ income, membership fees, and/or investments in gear, as determined by its members.	Catch is traded by fishbuyer or co-op and their capital increases. In our model a co-op gets 100% of the price offered by the market, and its members get all the money from the sales, but will then pay a membership fee to the co-ops’ capital. In addition, they will contribute small but significant amount of 0.0005% to the co-ops’ capital if their capital is above a certain threshold. This allows co-ops to accumulate capital and share with members in times of need.
6. Update fisher’s capital:	Loan is discounted from value of catch and resulting amount is fishers’ income. Co-op fishers get income as agreed by its members.	Fisher’s capital increases by the revenue of the landings, and the loan is deducted if the fisher did not cheat. If the fisher cheated he keeps the loan.
7. Renew Contract:	Contract is renewed between fishbuyers and fishers based on the ‘success’ of their interactions, and marked demand.	Fishbuyer keeps contract with fishers unless over supplied. If a fishbuyer’s capital is below zero the fishbuyer will go out of business, and his fishers become inactive. If a co-op member has cheated too much, he will have to leave the co-op due to low loyalty. If a co-op’s capital is below zero or the size of the co-op is too small, the co-op will go out of business.

There are two main differences between co-ops and PCs in the daily activities. First, because PCs have no formal membership contract, they can constantly ‘shop and select’ fishers from the fishery pool with the highest reliability, while co-ops are ‘stuck’ with their members whether they are reliable or not [61]. As such, PCs can attract members to meet their demand while co-ops cannot. Second, co-ops share written contracts defining entry and exit rules, and roles and responsibilities among members. Consequently, co-ops have higher transaction costs in establishing relationships. This is modeled as slower growth in loyalty for co-ops (see Table 1).

#### Annual entry dynamics of fishbuyers and co-ops

In addition to the daily activities, at the beginning of each year one fishbuyer and one co-op will enter the fishery. The fisher with the highest capital (chosen from the pool of inactive fishers) enters as a fishbuyer and forms a PC with five random inactive fishers. A co-op is also formed, and is constituted by five random inactive fishers. Empirically, in Mexico, we find that co-op formation is often linked to government incentives, which in turn are linked to new government formation every sixth year. However, we have chosen an annual entry scenario to be able to compare the number of co-ops and PCs in setting where both have equal possibilities of entering [6]. Note also that we do not explicitly consider any government involvement in the formation or management of co-ops or PCs, e.g. in form of regulations or subsidies. Each fishbuyer or co-op receives an equal and fixed share of the market when they enter. We keep these external conditions fixed and homogenous in order to be able to compare the effect of the micro-level dynamics for the success of the two organizational forms.

#### Fish population dynamics

The growth of the fish population is modeled using a single-species population model with logistic growth, and a linear harvesting term, following the well-established Gordon-Schaefer model [62], see S1 Calculations Eq 21. The fish market provides a fixed price for fish to the fishbuyers and co-ops, and is able to buy unlimited amounts of fish.

### Calibration, simulations and experiments

We calibrate the model so that the fishery can sustain 50 fishers and so that the income is equal to the expenses of each organization (S1 Calculations). In our simulations, when more than 50 fishers have entered, the fish stock will be over harvested, and organizations will start to go out of business. Because of the yearly entry, there is a constant pressure on, and competition for the resource, thus creating a dynamic fishery. We let the simulations run for 100 years (36500 time steps), to increase the possibility for the model to stabilize with respect to the average number of active fishers in each organization.

A specific setting of parameters and initial conditions in a simulation is termed an ‘experiment’. We analyze the experimental outcomes according to the number of fishers in each self-governance form (PC or co-op) as the average of the last 33 years. We chose this outcome variable because we are interested in emerging, quasi-stable patterns and not, the initial dynamics or small-scale yearly variations. Further outcome variables are i) the reason why co-ops and PCs go out of business, calculated as the accumulated sum of co-ops or fishbuyers that dissolved for a specific reason (e.g. lack of capital, loyalty, or members); and ii) the number of years each organization persists (counting the number of years from establishment to going out of business).

**Fig 4:**
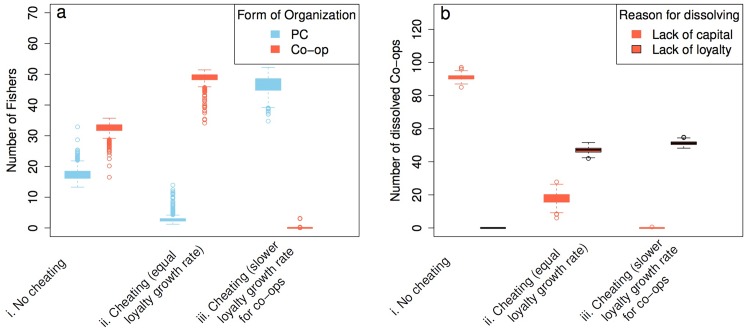
Model validation. **Panel a.** The number of fishers active in a PC or a co-op in the calibrated model but with annual entry dynamics (i) and different model assumptions (ii and iii). **Panel b.** The number of co-ops dissolving because of lack of capital or lack of loyalty among members to the co-op. Reliability and fishing skills are equal for all fishers (set to 1.0 and 0.5 respectively), and initial loyalty = 0 for all fishers. **Panel a** and **b** are based on the same data. The data represents an average of 500 repetitions.

**Fig 5:**
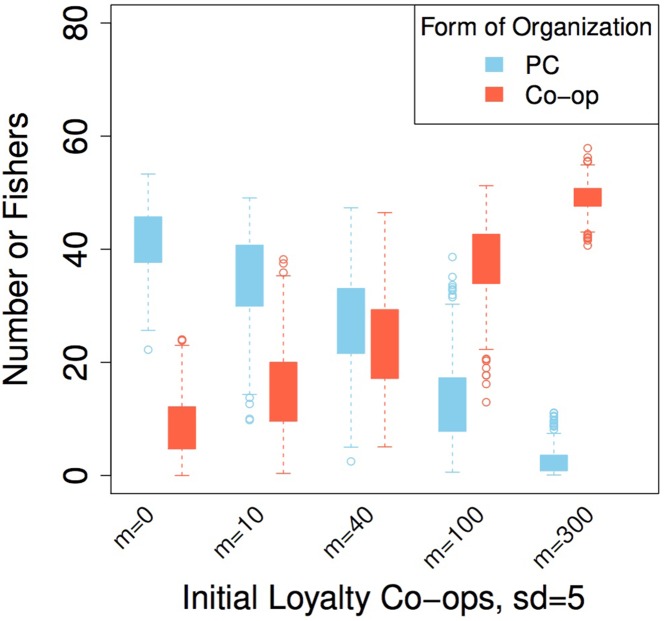
The effect of initial loyalty among fishers on the subsequent establishment of a co-op. The data represents an average of 500 repetitions.

**Fig 6:**
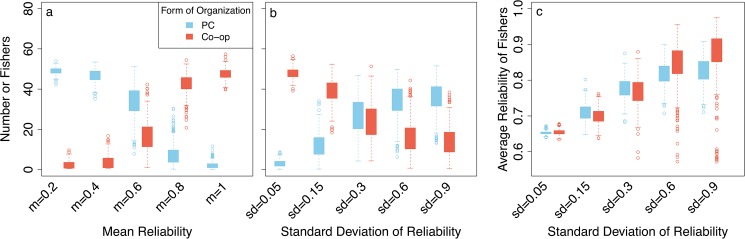
The effect of variation in fishers’ reliability. **Panel a**. The effect of the variation of mean reliability on fishes’ membership in PCs versus co-ops, sd = 0.3. **Panel b**. The effect of variation in standard deviations of reliability on fishers’ membership in PCs vs. co-ops (mean = 0.65). **Panel c**. The effect of variation in standard deviations of reliability on the average reliability of fishers in PCs or co-ops. Initial loyalty = 40, sd = 5. Data represents an average of 500 repetitions.

**Fig 7:**
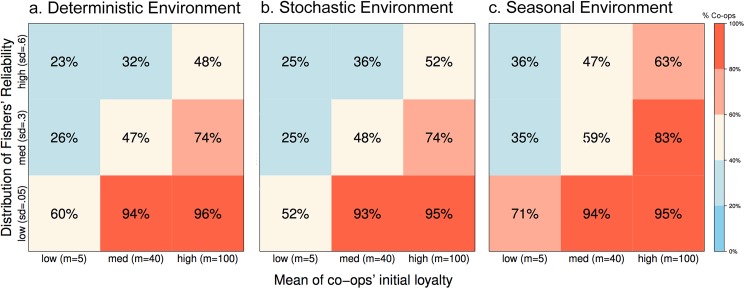
**Trends in the dominance of membership in co-ops versus PCs with varying initial loyalty for co-op fishers and varying distributions of fishers’ reliability under different environmental conditions (Panel a-c).** The numbers indicate the percentage of fishers’ membership in co-ops; red indicates co-op dominance, blue indicates that PCs are dominant, and beige indicates co-existence at roughly equal membership in each form. In the stochastic environment (**Panel b**), the catch is highly variable during the whole year, and in the seasonal environment (**Panel c**), catch varies following seasonal patterns. The data is based on an average of 3000 repetitions.

**Fig 8:**
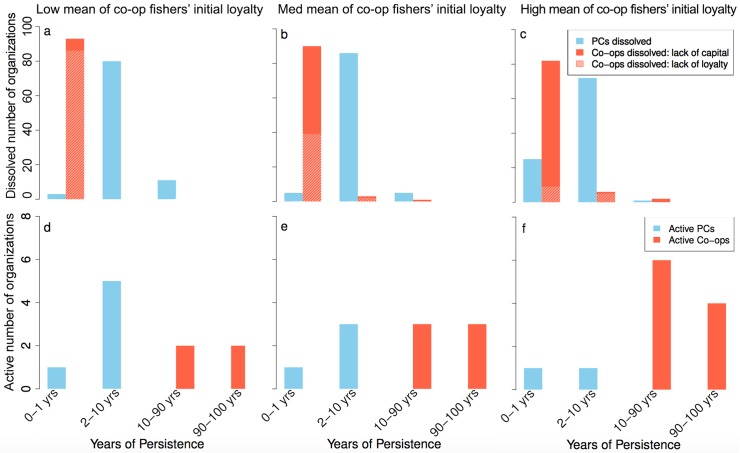
Years of persistence (age) of PCs and co-ops are dependent on co-op fishers’ initial loyalty (low, medium, high). **Panel a-c** shows the average age of an organization that dissolved during the simulation (100 years) and the reason why they dissolved (lack of capital or loyalty). **Panel d-f** shows the average age of the active organizations at the end of the simulation. The data is the same as for the “middle” horizontal section of Fig 7A (mean reliability = 0.65 (sd = 0.3), initial loyalty is 5 (low), 40 (medium) or 100 (high) with sd = 5 respectively, in a deterministic environment).

We designed the following experiments to answer our research questions (Table 3). Each experiment is repeated 500 (Figs 4–6) or 3000 (Figs 7–8) times depending on the level of stochasticity in the setup.

**Table 3:** Summary of experiments, variables tested and their range. Sd = standard deviation.

Experiment	Variable(s) tested	Range
**Initial Loyalty (Fig 5)**	Mean of initial co-op loyalty distribution (sd = 5).	Mean: {0, 10, 40, 100, 300}
**Variable Reliability (Fig 6)**	Distribution of reliability.	Mean: {0.2, 0.4, 0.6, 0.8, 1.0} (Fig 6A) Sd: {0.05, 0.15, 0.3, 0.6, 0.9} (Fig 6B and 6C)
**Interaction between initial loyalty and reliability (Fig 7A)**	Sd of reliability distribution (mean = 0.65), mean of initial co-op loyalty distribution (sd = 5).	Sd reliability: {0.05,0.3,0.6). Mean initial loyalty: {5, 40, 100}
**Environmental fluctuations (Fig 7B and 7C)**	Sd of reliability distribution (mean = 0.65), mean of initial co-op loyalty distribution (sd = 5). Stochastic: random fluctuations in catch rates (e.g. caused by changing weather conditions; S1 Calculations Eq 29). Seasonal: seasonal variation in stock abundance (S1 Calculations Eq 30).	Sd reliability: {0.05,0.3,0.6). Mean initial loyalty: {5, 40, 100}

For a detailed description of the model according to the ODD+D (Overview, Design concepts, and Details + human Decision-making) protocol [63,64], see S2 Table. The model was implemented in Netlogo [65] and analyzed using R [66].

## Results

### Validating the model

To gain confidence in the model, we start with a basic calibrated version and test model behavior by slowly introducing more complexity. The model is calibrated such that five co-ops and five PCs with identical loyalty growth rates and 50 active fishers with identical fishing skills and reliability can survive and the fishery will remain ‘sustainable’ (i.e., the fish stock is not over fished and fishers do not lose income; S1 Calculations). In this reference setting there is no cheating, the 50 fishers are active from the start of the simulation and no entry of other fishers, co-ops, or fishbuyers is possible.

When we add annual entry of PCs and co-ops, we find that co-ops have a better chance of surviving (Fig 4Ai). When cheating is introduced, most fishers are organized in co-ops (Fig 4Aii). PCs cannot survive because they accumulate less loyalty and hence cannot cope with high levels of cheating. It takes more time for PCs to build up loyalty because the fishbuyers regularly break the informal contract with fishers during the process of attracting and dismissing fishers. If a fishbuyer works with new fishers, there is no loyalty built up between them. Consequently, newly attracted fishers cheat more, causing the fishbuyer to lose capital faster than co-ops, making PCs go out of business before co-ops. The reason why co-ops might dissolve in this scenario is because they lack loyalty and not capital (Fig 4Bii). However, if the loyalty growth rate for co-ops is reduced to half, to reflect the higher initial transaction costs for initiating collective-action, we see a dominance of PCs (Fig 4Aiii). Here as well, lack of loyalty, not capital, constitutes the main reason for the dissolution of co-ops, as cheating causes loyalty to deteriorate faster than it is able to built up (Fig 4Biii). To illustrate the dynamics of the fishery over time we show an example simulation with only one repetition in Box 1.

**Box 1. An example of the interaction between fishers, loyalty, capital and the fish stock.**

To illustrate the dynamics of the model we zoom in to a one time-dynamic simulation example. Because of good initial conditions, co-ops and PCs are able to become established in this first 10 year phase. After about 15 years the stock has declined below the maximum sustainable yield (MSY) and fishers’ capital starts to decline (c, d). While the number of fishers that are active in co-ops stabilize after about 10 years, fishers active in PCs fluctuate as depicted by the two peaked curves (a). The loyalty and capital for co-ops increases over time, but the average loyalty for PCs fishers and the capital available to buyers fluctuates (b, c). After about 40 years the membership in PCs and co-ops stabilizes (a). Individual fishers’ capital is similar between PC and co-op fishers, however co-ops joint capital and loyalty is higher than PCs. Simulation settings: reliability mean = 0.65 and sd = 0, fishingskills mean = 0.5 and sd = 0, low initial loyalty.

#### The effect of a history of working together (initial loyalty)

From now on, and for the simulation experiments, we assume that the possibility of cheating is always present, loyalty accumulates at a half rate for fishers in co-ops and, the population of fishers is heterogeneous with respect to fishing skills (m = 0.5, sd = 0.3) and reliability (m = 0.65, sd = 0.3 or as set in the different experiments). Under these conditions PCs dominate the fishery if co-op members have no history of working together (initial loyalty). Only for an initial loyalty distribution with a mean of 40 and a standard deviation of 5, do co-op members perform equally well as those fishers in a PC, with 25 fishers organized in each of the two governance forms (Fig 5). For higher average initial loyalty of fishers, more fishers are organized in co-ops.

We now have a model that takes into account some of the empirical observation of the differences between PCs and co-ops: Fishers in the same co-op need some initial loyalty (a history of working together) to establish, while fishers in a PC do not. Co-ops suffer more from cheating members because this leads to a deterioration in loyalty between them (slower loyalty growth rate) and the co-op risks to go out of business.

### Model experiments

We now continue to deploy the model to investigate our research questions.

#### Variable reliability

Cooperative forms of self-governance can only dominate the fishery if the average reliability of fishers is high and, consequently, levels of cheating are low (Fig 6A). At the same time, co-ops are strongly affected by the degree of heterogeneity of fishers’ reliability and can only thrive in rather homogenous communities (Fig 6B). Taken together, co-ops and PCs coexist in about equal numbers at intermediate values of reliability (ca. 0.65) and lower levels of heterogeneity (sd = 0.3; Fig 6B). When heterogeneity is higher fishers are mainly organized in PCs. However, those in co-ops have higher reliability (Fig 6C). Co-ops can only establish and persist when the reliability of the fishers that join the co-op is high, which explains their higher average reliability. Fishbuyers, by contrast, can select for more reliable members, which enables them to thrive better in a highly heterogeneous community.

#### Interaction between initial loyalty and reliability

So far, we have separately investigated the influence of selected attributes of fishers or co-ops, namely their reliability or initial loyalty. Next, we examine the interactions between different levels of initial loyalty and different variance in reliability (Fig 7A). Fishers in co-ops dominate the fishery when there is high initial loyalty among their members and low variance in fishers’ reliability (Fig 7A) as already shown in Fig 6B. Membership in PCs dominates when there is high variance in fishers’ reliability and low initial loyalty among members of co-ops. PCs also dominate in instances where there exists medium to high variance of fishers’ reliability or low to medium initial loyalty among co-op’s members (upper left squares in Fig 7A). Membership in co-ops can only dominate when variance in fishers’ reliability is medium or low and initial loyalty is at least medium. At intermediate values for initial loyalty and reliability, equal memberships in PCs and co-ops are found as would be expected due to model calibration.

#### The influence of environmental variability

Compared to a static environment, co-op membership is slightly lower in a stochastic environment for low variability in fishers’ reliability, but marginally higher for high variability (Fig 7B). In a seasonal environment, however, the number of fishers organized in co-ops drastically increases for low initial loyalty and low distribution of reliability (from 60% to 71%), and further increases for medium and high reliability for all levels of initial loyalty (Fig 7C). Co-ops thrive better in a seasonal environment because they are able to accumulate slightly more capital, which serves as a buffer during a low season in the seasonal environment. In the stochastic environment however, there is no time for co-ops to build up this buffer capital and hence no large difference when the deterministic environment is observed.

#### The dynamics of establishment and persistence of forms of self-governance

We analyze the dynamics and reasons for establishment and failure of co-ops and PCs over the simulation period in order to understand the mechanisms and social-ecological interactions between fishers and the fish population that determines the dominance of each organizational form (Fig 8). We find that co-ops are more sensitive to low loyalty early on, and will go out of business because of a lack of loyalty among fishers within the first year (Fig 8A). This is earlier than for the majority of PCs, which go out of business only between the second to tenth year. Once initial loyalty increases (Fig 8B and 8C), lack of capital becomes the major reason for co-ops to go out of business, but the vast majority still dissolve within their first year of existence.

Co-ops that persist for a long period have generally established themselves early in the simulation when the fish stock was in a good condition and fishing pressure was not too high. These co-ops persevered through the hard times, while PCs have never persisted across the whole 100-year simulation (Fig 8D–8F). PCs tend to establish and persist longer than one year, but few make it past the 10-year threshold. Because fishbuyers generally enter with more capital, they will persist longer than co-ops, when entering during worse resource conditions. However, independent of when they enter the fishery, fishbuyers will take on more and more fishers to fulfill their demand in times of low stock abundance, which makes them lose capital because the fishers are not bringing in enough catch to pay back their loans.

## Discussion

We developed an agent-based model that incorporates and tests empirical understanding about the social and ecological factors and processes that are relevant for explaining the success of cooperative and non-cooperative forms of self-governance in SSFs in Northwest Mexico. The model allowed us to identify micro-level mechanisms that explain differences in fishers’ membership in two archetypical forms of self-governance: PCs and co-ops. We find that, all conditions being equal, a community is more likely to be dominated by co-ops if; fishers’ reliability is high on average, distributed rather homogeneously and, co-ops can build on previous cooperative experiences. High levels of reliability or (initial) loyalty within a co-op or between a fisher and a fishbuyer can be the result of social norms that prohibit cheating and encourage reciprocity. The importance of social norms for the performance of PCs or co-ops has been shown in SSFs in India and Kenya for instance [9,10]. In our model, it is ultimately the interplay between individual compliance and an emerging social norm at the community level that creates trust and stabilizes social interactions.

The two self-governance arrangements also clearly differed in terms of the number of years each organization persisted. Co-ops persisted through the complete simulated time period from the time they were established, while fishbuyers became established but then went out of business. Hence, fishers in co-ops are able to rely on long-term security for their fishing and trading operations, (as has been shown empirically for the Punta Allen co-op [47]), while fishers organized in PCs are exposed to more turbulent conditions for securing their livelihood. When exposed to environmental variability, co-ops were better equipped to handle seasonal variability, while both forms performed equally well under stochastic variability.

### Micro- to meso-level interactions affecting the emergence of co-ops and PCs

In our model, increased loyalty creates a positive (reinforcing) feedback loop, which leads to less cheating, which in turn increases loyalty. If members of a co-op are able to overcome initial transaction costs they are able to start building strong relationships within their organization because of the high entry and exit barriers for fishers’ membership. As a result, co-ops, once established, seem to be better equipped to cope with economic or resource variability caused by; overfishing, seasonal scarcity or other factors. This is in line with studies by McCay et al. [48] and Ovando [8]. Fishbuyers on the other hand, can easily adjust their fisher pool according to demand by dismissing fishers with the least loyalty and picking the ones with the highest reputation from the pool of available fishers. The frequent exchange of fishers, however, reduces their ability to build strong loyalty, which leads to higher levels of cheating, thus making it harder to accumulate capital over time and to cope with economic or resource variability.

Co-ops and PCs face income losses when fishers cheat and when the resource is scarce. Both types of organization continue to lend money to their fishers even though they are losing capital, i.e. when stock abundance is low. Thus, occasionally the buyers get ‘trapped’ by their own fishers because they keep lending money without recovering their loans, and, as a consequence, buyers will go out of business. Co-ops suffer from this feedback loop as well, but since co-ops cannot take on more fishers in challenging times, co-ops are less exposed to this phenomenon.

By unpacking the links between the micro and the meso-level in our model we can identify two key organizational phases for PCs and co-ops. In the establishment phase, the main challenge for co-ops is to overcome the initial transaction costs of devising working fishing and trading operations (a similar argument is also made by Townsend et al. [57]). Success during this initial phase is dependent on the composition of the co-op members, their history of working together and hence, their loyalty towards each other. Resource conditions during this time are also important. Only when these favorable conditions; high reliability among members, high initial loyalty and sufficient resource abundance coincide can the co-op establish itself. Once established, PCs and co-ops enter a second phase where they need to adapt to ever-changing conditions. In our model, the only option for success is to have accumulated more financial capital than the other PCs and co-ops. This makes it possible for the financially stronger PCs and co-ops to persist during low levels of stock abundance, as the financially weakest PCs and co-ops will exit first.

### Unraveling the interplay between fishers, organizations, and community level patterns using the agent-based model

Our results reveal an intriguing interplay between three levels; the fishing community (macro) level (the fish stock, the population of fishers, the patterns of Co-ops and PCs), the organizational (meso) level (a PC or a co-op), and the individual (micro) level (fishers’ reliability and loyalty). The community-level patterns of fishers’ reliability and loyalty influenced the cheating behavior and ultimately the organizational membership at the community-level. In turn, the organizational membership influenced the macro-level patterns determining the predominance of either co-ops or PCs in a community. Hence, it is the interplay between these three levels that explains the dominance of PCs or co-ops on the macro-level.

The agent-based model has allowed us to explore under what conditions and how, individual decisions and actions within a dynamic social and ecological environment give rise to cooperative or non-cooperative outcomes. This novel approach of combining synthesized insights and hypotheses from multiple empirical studies with agent-based modeling provided a means to unpack micro-level and cross-level dynamics that are difficult to measure in the field. Our explanation for the relative success of cooperative and non-cooperative forms of self-governance is one possible and plausible explanation of the observed empirical patterns. However, other explanations may be equally plausible because of the complexity and non-linearity of these systems. Whether or not our explanation remains a plausible and compelling explanation of the emergence of different self-governance forms in other parts of the world, and in different political and social contexts remains an open question for future research.

While agent-based models that investigate the dynamics of self-governance arrangements in small-scale fisheries are scarce, Janssen and Ostrom [37] show in their model that sustainable fishing can be achieved when actors belong to a stable group, there is no cost for developing trust, and the costs of monitoring and applying sanctions are low. Similarly to our study, their work emphasizes the importance of the initial composition of the group as foundation for successful collective action. Furthermore, Bravo [36] investigated the emergence of an institution, i.e. a shared harvesting rule, where actors can vote according to their beliefs about optimal harvesting levels. Ghorbani and Bravo [35] studied the evolution of a single institution in a theoretical model. Both these models are situated in a general common-pool resource setting, and do not incorporate trust nor PCs. Our focus on different dimensions of trust, the persistence of self-governance arrangements, and the pre-dominance of different forms of self-governance thus fills an important gap in current agent-based models.

### Model limitations

One of the constraints when building this model was the scarcity of data on micro-level processes such as cheating and lending, where we had to rely primarily on in-depth qualitative data from only two different sites. However, the identified variables align with the general literature on the role of trust for compliance in common pool resource management [13]. We see our model as a first step towards understanding micro-level explanations of self-governance, using Northwest Mexico as a starting point. We hope to stimulate other empirical researchers to test whether similar mechanisms may be at play in determining existing self-governance forms.

Modeling a complex social-ecological system to answer a specific research question necessarily entails leaving out many details. We have focused our exploration on different dimensions and the dynamics of trust resulting from social and social-ecological interactions as they have been hypothesized as important processes explaining the success of different self-governance forms. We have also focused on the persistence of each governance form and not their formation and thus we have necessarily neglected factors that are important in the formation process such as social class or social skills. Furthermore, the model does not allow co-op members to diversify their activities in order to adapt to changing environmental conditions, nor to expand their operations, nor are there mechanisms for long-term resource management. These options have been identified as important attributes of co-ops in contributing to their economic and ecological success [46,47,49,57]. By not including these options in the current model we make it even more difficult for co-ops to become established and persist.

A critical assumption influencing the interpretation of our results is that PCs and co-ops can form unconditionally. Globally, there are areas where strong institutions of patron-client relationships are already in place as in Kenya, Zanzibar or India for instance [9,10,12], or co-ops are incentivized by the government. In these contexts, the entry dynamics of co-ops need to be modified which is an interesting avenue for future research. As with the omission of alternative livelihood options for co-ops mentioned above, our decision for unconditional entry is justified, as the focus of our study is on the role of social interactions and resource dynamics. To what extent advantages provided to one or the other form through subsidies or historical conditions would increase their chance of establishment and persistence is a subject for further research.

We make the assumption that there is always an incentive to cheat and to keep prices fixed. Incorporating a fluctuating price mechanism between organizations would even out the overall effect of fluctuating prices over time, and this would not affect our results. However, interviews with fishbuyers highlight that when demand for a product goes up, cheating increases. This mechanism would be interesting to explore further by incorporating a fluctuating demand which would in turn impact prices, and thus allow the model to connect cheating to prices offered by other fishbuyers or co-ops.

## Concluding remarks

The combination of empirical research with agent-based modeling proved to be a productive and fruitful approach for exploring and testing micro-level explanations for differences in the success of cooperative and non-cooperative self-governance in SSFs in Mexico. This iterative, multi-methods approach of developing empirical hypotheses based on empirical data, expert knowledge, and literature and implementing them in our agent-based model has generated a more profound understanding of key mechanisms and the interplay between micro-, meso- and macro- institutional levels. The close cooperation between field experts and modelers was perceived by both groups as an enriching and productive experience, and a promising way forward to address complex real world problems in social-ecological systems.

International multi-lateral organizations like the World Bank [1] have identified the need to reform SSFs and are prepared to invest millions of dollars to do so. This paper provides a first step in addressing uncertainties regarding some of the factors that underpin how cooperative and non-cooperative self-governance can become established and persist. The results point towards the need to account for the levels of trust in a community and the heterogeneity of fishers when planning interventions to promote cooperative governance. However, more research is needed to understand the importance of micro-institutions in SSFs in different social and geographical contexts, and thus test the generalizability of our findings. With this study, we hope to stimulate further empirical work that tests these explanations and contributes to understanding the conditions under which the identified mechanisms hold.

## Supporting information

S1 Table Model assumptions and empirical justification. (PDF)

S1 Calculations Model calibration and parameterization, equations, and parameter settings. (PDF)

S2 Table ODD+D Protocol. (PDF)
