# Correction: Micro-level explanations for emergent patterns of self-governance arrangements in small-scale fisheries—A modeling approach

**DOI:** 10.1371/journal.pone.0179439

**Published:** 2017-06-06

**Authors:** The PLOS ONE Staff

Fig 9 does not appear in the article. In the sub-section “Validating the model” in the Results, the last sentence of the second paragraph should read: To illustrate the dynamics of the fishery over time we show an example simulation with only one repetition in Fig 9 as explained in Box 1.

**Fig 9:**
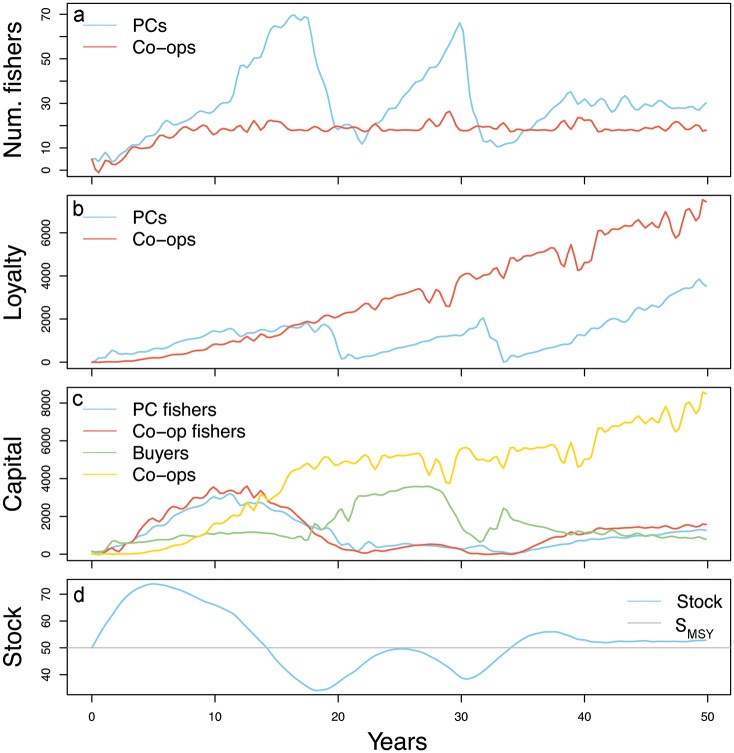
A time-dynamic simulation example illustrating how the number of fishers, loyalty, capital and stock size interact over time. **Panel a** shows the average number of fishers that are active in PCs or co-ops. **Panel b** shows the average loyalty of fishers in PCs or co-ops. **Panel c** shows the average capital of fishers in PCs, fishers in co-ops, buyers, and co-ops. **Panel d** shows the stock size, and the stock size that would result in the maximum sustainable yield (S_MSY_).

In Box 1, the first sentence should read: To illustrate the dynamics of the model we zoom in to a one time-dynamic simulation example (Fig 9).

Please view Fig 9 here. The publisher apologizes for this error.
