# Supplementary material for: Micro-level explanations for emergent patterns of self-governance arrangements in small-scale fisheries—A modeling approach
Source: PLoS One. 2017 Apr 13;12(4):e0175532. doi: 10.1371/journal.pone.0175532 (PMC5391074; doi:10.1371/journal.pone.0175532)
Supplement: S1 Table — (PDF) [file pone.0175532.s001.pdf]

S1 Table. Model assumptions and empirical justification

S1 Table. Justification of assumptions and model interpretation, complement to Table 1 and 2 in the main paper. Interviews and observations were made by X. Basurto.

Activity	Empirical Justification	Model interpretation
Buyer enters	Buyers are former fishers or outsiders with capital that move to the community with the purpose of commercializing fish. Fishers turned fishbuyers have access to capital or as stated in interviews "are good with numbers" or "good with people."<br><br>Source: interviews	The inactive fisher with the highest capital turns buyer and enters the fishery as a buyer
Co-op forms	Commonly co-ops form by fishers who are related by kinship or friendship, and thus are very likely to have a joint history of working together. The law in Mexico says a minimum of six people should form a co-op, however in the model five is used for calibration reasons.<br><br>Source: interviews and literature [1–6]	Five random inactive fishers form a co-op, and share the same initial loyalty to each other.
Buyer stops working with a fisher	There will be times when the buyer will not need all its fishers, for example when the market is saturated and demand is low. The buyers will commonly stop working with the least reliable fishers.<br><br>Source: interviews	If a buyer is over supplied with fish, he stops working with the fisher whom he has built up least loyalty
Update fish population	Standard simple population dynamics commonly used in bio-economic fisheries models<br><br>Source: literature [e.g. 7]	Fish stock grows according to internal growth rate and actual fishing mortality, in some modeling scenarios influenced by environmental variability

Cheating	Fishers often face incentives to cheat because a higher price is offered for their catch by a different fishbuyer, or for other reasons. Fishers that cheat to their fishbuyer or co-op are described by the fishbuyer/co-op as having low reliability and little loyalty to them.<br><br>Source: observations and interviews	Whether a fisher will cheat on his fishbuyer or co-op (and land his catch to another fishbuyer or co-op) depends on the fisher's reliability and his loyalty to his fishbuyer or co-op. We assume that there is always a reason to cheat and thus do not link it to any price mechanism, the reason being that we want to analyze different dimensions of trust in relation to cheating.
Buyers go out of business	Interviewees mentioned debt accumulation as the main reason for fishbuyers' leaving the business.<br><br>Source: observations and interviews	Buyers exit when their capital is less than zero
Co-op goes out of business	Commonly co-ops go out of business because of lack of capital or lack of loyalty.<br><br>Source: observations and interviews	Co-ops go out of business because their capital is less than zero, or they have to few members because members with low loyalty were dismissed.
Update loyalty	Co-ops need to engage in collective action while PCs have relationships with individual fishers. Hence, we assume the transaction costs to achieve collective action are higher, and loyalty grows more slowly among members of a fishing co-op.<br><br>Source: observations and interviews	If a PC or co-op fisher cheats, loyalty decreases by one, if a PC fisher does not cheat his loyalty increases by one, while the loyalty of a non-cheating co-op fisher only increases by 0.5.
**Feature**	**Empirical Justification**	**Model Interpretation**
Co-op size	A written contract is required for co-ops to form. Entry and exit of	Co-ops can't grow and have five fixed members from the start.

	new members to the organization requires voting and other formal written procedures making it a slow process.<br><br>Source: observations and literature [2]	
PC size	Fishbuyers and fishers informally contract and so entry and exit of fishers to this form of organization is fast. Fishbuyers explain that the constant rotation of their fishers is an important activity occupying their time. Commonly fishers are dismissed and attracted within a time frame of one to three weeks.<br><br>Source: observations and interviews	PC can grow and shrink its crew between 1-15 fishers. The buyer can dismiss a fisher every 15 days (starting at day 15), and attract a new fisher every 15th day (starting at day 18, so there is a delay of 3 days before the buyer can attract a new fisher).
Market demand	Source: observations	Market demand is infinite
Co-op fishers price	Well established co-ops sell directly to the market, and co-op don't need pay anything to a specific buyer that brings their catch to the market.<br>Source: literature [8]	Co-op fishers get 100% of market price
PC fishers price	Fishbuyers sell to the market, and take a share of the profit before paying their fishers.<br><br>Source: literature [8]	PC fishers get 80% of market price
Co-op membership fee	Co-ops set their own rules for membership fees.<br><br>Source: Literature [9]	Co-op fishers pay a small fee 0.0005% to their co-op if his capital is above a certain threshold.
The pool of fishers the buyer can choose from	Buyers cannot know every fisher in the fishery because not all will belong to the community.	The buyers can only know the reliability of maximum 25 fishers in the community. 25 is roughly based on interviews with fish buyers but

	Source: observations	ultimately chosen arbitrarily for modeling purposes.

1. Basurto X, Bennett A, Weaver AH, Rodriguez-Van Dyck S, Aceves-Bueno J-S. Cooperative and Noncooperative Strategies for Small-scale Fisheries' Self-governance in the Globalization Era: Implications for Conservation. Ecology and Society. 2013;18: 38.

2. Bennett A. The influence of neoliberalization on the success and failure of fishing cooperatives in contemporary small-scale fishing communities: A case study from Yucatán, Mexico. Marine Policy. 2016;

3. McCay BJ, Micheli F, Ponce-Díaz G, Murray G, Shester G, Ramirez-Sanchez S, et al. Cooperatives, concessions, and co-management on the Pacific coast of Mexico. Marine Policy. 2014;44: 49–59.

4. Méndez-Medina C, Schmook B, McCandless SR. The Punta Allen cooperative as an emblematic example of a sustainable small-scale fishery in the Mexican Caribbean. Maritime Studies. 2015;14: 1–19.

5. Cinti A, Shaw W, Cudney-Bueno R, Rojo M. The unintended consequences of formal fisheries policies: social disparities and resource overuse in a major fishing community in the Gulf of California, Mexico. Marine Policy. 2010;34: 328–339.

6. Soberanes Fernández JL. Historia contemporánea de la legislación pesquera en México. El regimen jurídico de la pesca en México. 1994; 1–26.

7. Clark CW. Mathematical bioeconomics: the mathematics of conservation. 3rd ed. Wiley; 2010.

8. Bennett A, Basurto X. Local Institutional Responses to Global Market Pressures: The Sea Cucumber Trade in Mexico. Working Paper Series Duke Marine Lab Beaufort NC. 2016;

9. Basurto X. Finding Order Amid Complex Small-Scale Fisheries Self-Governance Arrangements [Internet]. Rochester, NY: Social Science Research Network; 2016. Report No.: ID 2873306. Available: https://papers.ssrn.com/abstract=2873306
