# Supplementary material for: Micro-level explanations for emergent patterns of self-governance arrangements in small-scale fisheries—A modeling approach
Source: PLoS One. 2017 Apr 13;12(4):e0175532. doi: 10.1371/journal.pone.0175532 (PMC5391074; doi:10.1371/journal.pone.0175532)
Supplement: S1 Calculations — (PDF) [file pone.0175532.s002.pdf]

S1 Calculations. Model calibration and parameterization, equations, and parameter settings

First we show the calibration and parameterization of the model that is made prior to the simulations. Next we explain the key functions in the simulation model. Finally we present a table of fixed parameters, their descriptions and values.

Calibration and parameterization

In this section we calibrate the parameters individual effort (e) and the market price ($marketprice$) to satisfy the following two conditions:

1) The fishery is sustainable when 50 fishers are actively fishing. We define sustainable as keeping the fish stock at the level, which maximizes the yield over an infinite time horizon i.e. maximum sustainable yield (MSY).
2) The income of an organization (PC or co-op) equals its expenses, and the income of a fisher equals its expenses.

We also use this as reference scenario to set the following constants in the simulations; $marketshare$, *demand, price, dailyloan, dailycostoffishing, dailyconsumptionfisher, coopfee.*

For the calibration we assume that there are ten active organizations. The organizations operate under 'ideal conditions' (fishers will not cheat), and fishers are identical with respect to fishing skills and reliability. We let the subscript MSY represent variables in the simulation that are only kept fixed during this calibration. Those parameters that lack subscript MSY are the constants we set in this calibration as listed above. We set the active fishers at calibration to be $numFishers_{MSY} = 50$, $numOrganizations_{MSY} = 10$, $organizationSize_{MSY} = 5$, and let the parameters of the fish population carrying capacity $K = 100$, and growth rate $r = 0.001$. Because of the logistic growth model used to represent the dynamics of the fish stock, the optimal stock size for a sustainable fishery is $K/2$, and the fishing effort that will keep the stock at the MSY is $r/2$.

The individual fishing effort that will keep the stock at the MSY is then calculated as

$$e = \frac{r}{2} \times \frac{1}{numfishers_{MSY}} \quad (1.)$$

Next, we find the market share and the demand. The total catch at MSY is

$$catch_{MSY} = \frac{K \times r}{4} \quad (2.)$$

The catch at MSY per organization (subscript o) is then

$$catch_{MSY,o} = \frac{catch_{MSY}}{numfishers_{MSY}} \times organizationSize_{MSY} \quad (3.)$$

We assume that fishbuyers and co-ops are satisficers, i.e. they do not aim to maximize profit but aim to catch the amount of fish that corresponds to their share in the market, which is set as

$$marketshare_o = \frac{organizationSize_{MSY}}{numfishers_{MSY}} \quad (4.)$$

This means that if more than 10 organizations are active the resource will be overharvested and if less than 10 organizations are active there will be under harvesting.

The demand of an organization, i.e. the amount of fish they want their fishers to catch (independent of current stock conditions), is

$$demand_o = marketshare_o \times catch_{MSY} \quad (5.)$$

Next we balance the income and expenses of the organizations. We first set a constant representing the sum of daily expenses for a buyer to $dailyconsumptionbuyer = 5$. This constant is used both in the calibration and the simulations. The buyer gives 80% ($\eta = 0.8$) of their income of the traded catch to the fishers. We calculate the fixed market price such that the buyer can balance its expenses and income, i.e. $dailyconsumptionbuyer = marketprice \times (1 - \eta) \times catch_{MSY,o}$.

$$marketprice = \frac{dailyconsumptionbuyer}{(1-\eta)\times catch_{MSY,o}} \quad (6.)$$

We also set the price given by a PC to a fisher as

$$price_{PC} = \eta \times marketprice \quad (7.)$$

The co-op does not take any profit, hence the price given by a co-op to a fisher is set to

$$price_{coop} = marketprice \quad (8.)$$

The price is thus different depending on which organization that provides it.

Next we calibrate the income and expenses of a fisher to be balanced at ± 0 as follows:

The income of a PC fisher

$$incomefisher_{MSY,PC} = catchfisher_{MSY} \times price_{PC} \quad (9.)$$

The income to a co-op fisher

$$incomefisher_{MSY,coop} = catchfisher_{MSY} \times price_{coop} \quad (10.)$$

Here the fishers' catch is calculated as $catch_{MSY}/numFishers_{MSY}$. In the actual simulations the catch depends on factors as well, see eq. 20.

The daily loan proportion of the total daily income and expenses of a fisher is set to λ = 0.15 of the income corresponding to the income of the PC fisher. The daily loan is the same for both PC and co-op fishers.

$$dailyloan = dailycostoffishing = \lambda \times incomefisher_{MSY,PC} \quad (11.)$$

The daily cost of fishing is thus what the fisher will borrow on a daily basis from its organization, and what the organization will lose if the fisher cheats. The remaining income is spent by the fisher as 'daily consumption' and is represented by the constant

$$dailyconsumptionfisher = (1 - \lambda) \times incomefisher_{MSY,PC} \quad (12.)$$

Hence, for a PC fisher the sum of the expenses, here the daily cost of fishing (eq. 11) and the daily consumption (eq. 12), equals the fishers income.

The coop fishers have the same daily loan and daily consumption, but have a higher income. The coop fee is thus set to balance the gap between the higher income of a co-ops fisher as co-op fishers share the same basic expenses as a PC fisher

$$coopfee = incomefisher_{MSY,coop} - dailyconsumptionfisher - dailycostoffishing \quad (13.)$$

Thus the coop fee also corresponds to the fishbuyers' profit. The coop fee is always paid even when the fisher cheats.

We have now set the constants that we will use in the simulations. Below we define the key equations for the simulation model, and those variables represented by the MSY subscript are now extended to include time dependent dynamics.

Simulation Equations

Functions that are modeled in the Netlogo model, and the equations they represent. Let $t = one\ time\ step$.

buyer-over-under-supplied: A buyer attracts or dismisses fishers according to its demand and the amount of catch brought by the fishers that the buyer is working with. A buyer is oversupplied if

$$(demand_o - catch_{o,t}) < 0 \quad (14.)$$

A buyer is undersupplied if

$$(demand_o - catch_{o,t}) > 0 \quad (15.)$$

Where $catch_o$ is the sum of the catch of the fishers the buyer is currently working with.

take-and-give-loan: Let index i represent a fisher, index j a buyer or a co-op, $N =$ the number of fishers working with the buyer or co-op, then the equations for calculating the capital of the actors, buyers and co-ops when the loan is taken in the beginning of the day (a time step), are as follows:

$$capital_{t+1,i} = capital_{t,i} + dailyloan + additionalloan_{t,i} \quad (16.)$$

$$buyercapital_{t+1,j} = buyercapital_{t,j} - \sum_{i=1}^{N}(dailyloan + \ additionalloan_{t,i}) \quad (17.)$$

$$coopcapital_{t+1,j} = coopcapital_{t,j} - \sum_{i=1}^{N}(dailyloan + \ additionalloan_{t,i}) \quad (18.)$$

go-fish: Active fishers go fish.

Let e = individual effort per fisher (eq. 1), N =total number of active fishers. Then the total fishing effort, *E,* is calculated as:

$$E_t = \ N_t \times e \quad (19.)$$

The catch of an individual fisher is calculated as

$$catch_{t,i} = \ 2 \times f_i \times \ e \times q_t \times s_t \quad (20.)$$

where , q = catchability (Note that q depends on t only for the stochastic scenario, figure 7b in the main manuscript, in the other scenarios q is constant and set to 1.0), f = fishing skills.

update-stock: Each time step the stock is updated to account for growth and harvesting. Let r = growth rate, K = carrying capacity, then the stock *s*, is updated as

$$s_{t+1} = r \times s_t \left(1 - \frac{s_t}{K}\right) - E_t \times q_t \times s_t \times \sum_{i=1}^{N} f_i \quad (21.)$$

Initial stock size is set to $K/2$.

cheat: If the reliability of a fisher is larger than a random number α, and $loyindex$ = $[0 \ldots 1]$ is less than an other random number β (random numbers between zero and one) and third, there is an other organization to sell catch to, the fisher will cheat. If loyalty of a fisher is above 365 there is only a 5% chance of cheating because of loyalty (see eq. 28 for how loyalty is calculated). Let $y = 365$, and $\theta = 0.05$, then, if loyalty of a fisher is < 365, the loyalty index is calculated as below, else $loyindex \ = \theta$

$$loyindex_{t,i} = \theta + \frac{y - loyalty_{t,i}}{y} \quad (22.)$$

$$if \ reliability_i > \alpha \ AND \ loyindex_{t,i} < \beta \ then \ cheat \quad (23.)$$

sell-catch: The net income of a fisher is its catch × price. The daily consumption is discounted from the fisher's capital. Fishers that did not cheat pay back their loan ($\gamma = 1$), but fishers that cheated do not return the loan ($\gamma = 0$). Let loan be the sum of the daily loan plus the potential additional loan, and price set according to eq. 7 or eq. 8 depending on who the fisher sells it catch to, then the capital is calculated as

PC fisher: $$capital_{i,t+1} = capital_{i,t} + catch_{i,t} \times price_{o(i,t)} - dailyconsumption - \gamma \times loan_{i,t} \quad (24.)$$

Co-op fisher: $$capital_{i,t+1} = capital_{i,t} + catch_{i,t} \times price_{o(i,t)} - dailyconsumption - \gamma \times loan_{i,t} - coopfee \quad (25.)$$

The price given thus translates to the following: if a fisher belongs to a PC and cheated with another fish buyer, the fisher will get the same price as the fisher would if not cheating. The same holds for a co-op fisher cheating to another co-op. If a PC fisher cheats with a co-op the fisher will get a higher price because co-ops pay more. If a co-op fisher cheats with a fish buyer he will get less because a fish buyer pays less. All cheating fishers keep the daily loan.

trade: The net income of a buyer is its $fleetcatch_{t,j} \times (1-\eta) \times marketprice$. The buyers' net income is added to its capital and the daily consumption is discounted. If the buyer has no fishers working for him, the daily consumption is still discounted. The fishers pay back their loans if they have the capital to do so.

$$buyercapital_{j,t+1} = buyercapital_{j,t} + fleetcatch_t \times (1-\eta) \times marketprice + \sum_{i=1}^{N} (\gamma_{i,t} \times loan_{i,t}) - dailyconsumptionbuyer \quad (26.)$$

Where the $fleetcatch$ is the sum of the fishers' catch that are working for him, but also including potential extra catch from cheating fishers that sell their catch to him. The loans are what the fish buyer has lent to fishers, $\gamma = 0$ if cheated else 1. The coops capital is updated as

$$coopcapital_{j,t+1} = coopcapital_{j,t} + \sum_{i=1}^{N} (\gamma_{i,t} \times loan_{i,t} + coopfee) \quad (27.)$$

coop-dissolve: the co-op will dissolve if 1) the aggregated loyalty of the co-op is less than zero, 2) the capital of the co-op is less than zero, and, 3) the size of the co-op is less than 3 members.

buyer-exit: A buyer will exit if its capital is less than zero. The buyer will turn actor, and thus a new fisher will be formed from this buyer with new characteristics and values. The loans of its fishers will be reset to zero.

update-loyalty: The loyalty of fishers that did not cheat increases 0.5 for co-op fishers, and for PC fishers by 1.0.

$$loyalty_{i,t+1} = loyalty_{i,t} + \omega_i \times \gamma_{t,i} - 1 \quad (28.)$$

Where $\gamma = 0$ if cheating occurred, and $\gamma = 1$ if no cheating occurred, and $\omega = 1.5$ for coop fishers and $\omega = 2.0$ for PC fishers. The last term, –1, thus reduces loyalty when cheating occurred.

fisher-exit: A coop fisher will exit the coop if its capital or loyalty is below zero. A PC fisher will only exit if its buyer goes out of business. Exiting the fishery means the fisher becomes

inactive and cannot fish, however it is still possible to enter the fishery again though another fishbuyer or through forming a new co-op.

stochastic-environmental-change:

The catchability q is affected by weather fluctuations affecting the possibility to go or find fish. Let q change uniformly within every 7th to 21st time step, then q is updated as

$$q_t = normalRandom(\mu, \sigma) \quad (29.)$$

Where $normalRandom$ represent that q is drawn from a normal random distribution with the mean $\mu = 1.0$ and the standard deviation $\sigma = 0.9$. $q = 1.0\ at\ t = 0$.

seasonal-environmental-change:

The change in the seasonal environment is represented as every 6 months some units of the biomass is removed and every other 6 months some units of stock is added. Let $\upsilon = 0.4$, then

$$Every\ half\ year\ starting\ at\ t = 182 \quad (30.)$$

$$s_{t+1} = s_t - K \times \upsilon$$

$$Every\ half\ year\ starting\ at\ 364$$

$$s_{t+1} = s_t + K \times 2\upsilon$$

Table 1 Parameters, descriptions and values.

Parameter	Description	Value
	**Fish Population**	
r	Growth rate	0.001
K	Carrying capacity	100
q	Catchability	1.0
	**Capital**	
λ	Share of daily consumption that is borrowed for gas and lunch. Defines what the fishers borrow daily, and what the buyer or co-op looses if cheating occurs.	0.15

$dailyconsumption buyer$	Daily consumption buyers	5.0
$initialcapital$	Initial capital of actors.	$dailyconsumptionfisher \times \varphi$
$initialcapitalbuyer$	Initial capital of buyer (maximum capital of x or y as defined in value/range column),	$max\ (dailyconsumptionbuyer \times \phi, capital\ when\ entering)$
$initialcoopcapital$	Initial capital for a new co-op, approximately equal to buyers initial capital	$organizationSize \times dailyconsumptionfisher \times \chi$
φ	For calculating $initialcapital$ for fishers. Fishers can survive one week without income or taking loan.	7
ϕ	For calculating $initialcapitalbuyer$. Fishbuyers can survive one month without income and not giving loans.	30
χ	For calculating $initialcoopcapital.$ Calculated to match fishbuyers survival without income.	9
	**Fishery**	
N_{tot}	Total number of actors (fishers and buyers).	100
