# Supplementary material for: Micro-level explanations for emergent patterns of self-governance arrangements in small-scale fisheries—A modeling approach
Source: PLoS One. 2017 Apr 13;12(4):e0175532. doi: 10.1371/journal.pone.0175532 (PMC5391074; doi:10.1371/journal.pone.0175532)
Supplement: S2 Table — (PDF) [file pone.0175532.s003.pdf]

S2 Table. ODD+D Protocol

Abbreviations: PCs = patron-client relationships, Co-ops = fishing cooperatives, SSF = small-scale fisheries, Buyer (fish-buyer). Definition: Inactive fishers are fishers looking for a buyer or a co-op. Active fishers are fishers that are already in a PC or co-op relationship.

Table S2. ODD+D protocol including guiding questions, examples and column for own model description, bold font denotes newly developed parts compared to the original ODD protocol [1].

		Guiding questions	Examples	Model description
**I) Overview**	I.i Purpose	I.i.a What is the purpose of the study?	Research question incl. test of hypothesis, **system understanding, theory development, quantitative predictions, management or decision support, communication and learning (participatory modeling)**	a) What is the effect of micro-level factors related to trust —such as fishers' reliability, and loyalty between fishbuyers and fishers and between members in co-ops—for the establishment and persistence of different self-governance arrangements? b) How does environmental variability affect whether co-ops or PCs will emerge as the dominant form of self-governance? c) How stable are these two self-governance arrangements and what makes them fail? The focus is on increased system understanding.
		**I.ii.b For who is the model designed?**	**Scientists, students/teachers, decision makers, stakeholders**	For scientists, particularly those interested in self-governance in social-ecological systems. For decision-makers to illustrate how e.g. social attributes can influence whether cooperative vs. non-cooperative forms of self-governance can establish and persist.
	I.ii Entities, state variables, and scales	I.ii.a What kinds of entities are in the model?	Agents / individuals (humans, institutions): types and subtypes, spatial units (grid cells), environment, collectives (groups of agents)	- two types of human agents: Fishers and fish-buyers, fishers can be members in either a PC or a co-op organization.<br>- one biological entity: a renewable resource of fish.<br>- one economic entity: fish-market with fixed parameters.<br>The simulation is non-spatial.

		I.ii.b By what attributes (i.e. state variables and parameters) are these entities characterized?	<u>Of Agents:</u> identity number, age, sex, maximum age, memory, location, level of resources, **ownership of land,** (political) opinion, occupation, **decision model (only mention the name of the strategy, which is explained later on), one agent represents one individual / one household / one farm / all individuals of one specific type**,<br><br><u>of spatial units</u>: location, a list of agents in a cell, **land owned by farmer**, descriptor of environmental conditions (elevation, vegetation cover, soil type), current land use<br><br><u>of collectives</u>: list of agents, specific actions<br><br>Units of measurement	ALL AGENTS: capital, effort, fishingskills, reliability, coopid, loyalty, revenue. FISHERS have in addition: fishedcatch,, loan, primarybuyer, secondarybuyer (the secondary buyer is the buyer a cheating agent will sell its catch to), decision-model based on pro-social preferences (loyalty). FISH-BUYERS have in addition to agent attributes: price, buyermarketshare, buyerdemand, fleetcatch, decision-model is "satisfying" (maintain current income level, risk averse). WHOLESALERS (THE MARKET) have in addition to agents: price<br><br>FISH_POPULATION: growth rate, carrying capacity, biomass.<br><br>Co-ops: coopcapital, cooployalty, coopmarketshare, coopdemand, coopsize
		**I.ii.c What are the exogenous factors / drivers of the model?**	**Disease, climate, lake water level, land cover change, tectonic disturbances, invasive species, legislation**	Exogenous: fluctuating catchability of fish stocks, seasonal dynamics of fish stock
		**I.ii.d If applicable, how is space included in the model?**	**Not included, spatial implicit, spatial explicit, georeferenced (GIS)**	Not included.
		I.ii.e What are the temporal and spatial resolutions and extents of the model?	One time step represents one year and the simulations were run for 100 years, one grid cell represents 1 ha and the model landscape comprises 1000 x 1000 ha	One time step is one day. The simulations were run for 100 years.
	overvie w and	I.iii.a What entity does what, and in what order?	Self-explanatory names of the model's processes, **including decision making**	**EVERY 1 YEARS:**<br><br>Global level: form-coop (one co-op forms),

			**processes,** pseudo-code of the schedule, synchronous / asynchronous update	fisher-turns-buyer (the fisher with highest capital turns buyer), set-shares-demand-price (a fixed market share is given to each buyer and co-op, this is also their demand. The buyers demand is what they strive to meet in supply from their fishers. The price given to fishers by the buyer or co-op is fixed (co-ops give market price, fish buyer give 80% of the market price).<br><br>**DAILY (every time step):**<br><br>See main paper a flow chart figure.
**II) Design Concepts**	**II.i Theoretical and Empirical Background**	II.i.a Which general concepts, theories or hypotheses are underlying the model's design at the system level or at the level(s) of the submodel**(s) (apart from the decision model)?** What is the link to complexity and the purpose of the model?		- Main model: common pool resource theory [2], principal-agent theory [3]<br><br>- Sub model (the biological entity): Gordon-Schaefer Model for fisheries [4]
		**II.i.b On what assumptions is/are the agents' decision model(s) based?**	**<u>Established theories</u> (micro-economic models: homo oeconomicus, full / bounded rationality; cognitive models: social psychology, mental models; space-theory based models)**<br><br>**<u>real-world observations</u> (mechanistic explanations / process-based understanding available; black-box, use of heuristics, statistical regression methods)**<br><br>**<u>ad-hoc rules</u> (dummy rules, e.g. constancy assumption)**<br><br><u>combinations</u> **of theory and observations**	Fishers decision-model on whether to cheat on their fishbuyer or not and land and sell their catch to another buyer is based on empirical observations.<br><br>Fishbuyers decide which fisher to work with based on empirical observations.<br><br>Co-op members will decide to kick out a member if this person is not fulfilling the assigned undertakings, based on empirical observations.<br><br>See S1 Table for a complete table linking each activity in the model to the empirical justification. Table 1 and 2 in the main paper.

		**II.i.c Why is a/are certain decision model(s) chosen?**	**Data (non-) availability, pattern-oriented modeling, reference to other studies, theoretical considerations**	Empirical findings have shown the importance of social attributes such as reputation and loyalty for fishbuyer and fishers in selecting whom to work with. [See e.g., 5–7]
		**II.i.d If the model / a submodel (e.g. the decision model) is based on empirical data, where does the data come from?**	**Participatory approaches (role playing games), household surveys, interviews, direct observations, statistical census, archives, GIS, field or lab experiments**	individual interviews with buyers and fishermen, direct observations, log-book data over 5 years from the diary of a main fishbuyer in Kino, Mexico, interviews with co-ops. [See e.g., 5–7]
		**II.i.e At which level of aggregation were the data available?**	**Household / individual level, group level**	Buyers: Individual level<br>Fishers: individual level<br><br>Co-ops: group level
	**II.ii Individual Decision Making**	**II.ii.a What are the subjects and objects of decision-making? On which level of aggregation is decision-making modeled? Are multiple levels of decision making included?**	**Name subjects (individuals agents / households, on communal level, top down decision maker) and objects of decisions, e.g.: Form of land use, distribution of labor, choices of buying and selling**	PC: Individual fishers decide which buyer to work with. Second, they also decide weather to cheat this buyer, and sell catch to a different buyer or co-op, which affects loyalty between them.<br><br>Co-ops: Individual fishers can cheat and decide to sell their catch to another co-op or buyer. Decision-making only occurs at the individual level.
		**II.ii.b What is the basic rationality behind agents' decision-making in the model?** Do agents pursue an explicit objective or have other success criteria?	**Rational choice (classical optimization approach, utility maximization), bounded rationality (satisficing approach), no objectives (routine based, trial and error)**	Buyers are satisfiers, i.e., they want to fulfill their market share but not more.<br><br>Buyers choose fishbuyers based on social attributes and fishing skills (their reputation as fishers), and stop working with them based on the loyalty developed between them. The objective is to get the most reliable and loyal fishing crew.
		**II.ii.c How do agents make their decisions?**	**Decision tree, utility function, random choice**	Decision is based on a set of rules:<br><br>Buyers decide on what fisher to work with (takes the fisher with maximum reputation).

				Fisher decides to cheat based on loyalty and reliability. See S1 Calculations.
		**II.ii.d Do the agents adapt their behavior to changing endogenous and exogenous state variables? And if yes, how?**	**Adaption of resource extraction level in dependence of ecological state of resource**	No.
		**II.ii.e Do social norms or cultural values play a role in the decision-making process?**	**Cultural norms, trust**	Yes, trust is incorporated though loyalty that forms over time.
		**II.ii.f Do spatial aspects play a role in the decision process?**	**Space-theory based models**	No.
		**II.ii.g Do temporal aspects play a role in the decision process?**	**Discounting, memory**	Yes though the loyalty that forms between fishers and buyers, and fishers in a co-op.
		**II.ii.h To which extent and how is uncertainty included in the agents' decision rules?**	**Not at all / stochastic elements mimic uncertainties in agents' behavior / agents explicitly consider uncertain situations or risk**	Not included.
	II.iii Learning	II.iii.a Is individual learning included in the decision process? How do individuals change their decision rules over time as consequence of their experience?	**Change of aspiration levels depending on past experiences**	No learning is included. However, loyalty changes over time and affects the decision.
		**II.iii.b Is collective learning implemented in the model?**	**Evolution, genetic algorithms**	No.
	**II.iv Individual** Sensing	II.iv.a What **endogenous** and **exogenous** state variables are individuals assumed to sense and consider in their decisions? **Is the sensing process erroneous?**		None.
		II.iv.b What state variables of which other individuals can an individual perceive?	**(Multiple) resources (including working power, monetary resources,**	Buyers know the exact reliability of maximum 25 inactive fishers when the buyer is deciding on which new fisher to

		**Is the sensing process erroneous?**	**other income resources) and behavior of other agents**	select. Hence the buyer cannot sense the reliability of all inactive fishers at a time. No information fishers or buyers can sense is erroneous.
		II.iv.c What is the spatial scale of sensing?	Local, network, global (whole model space)	Individual
		II.iv.d Are the mechanisms by which agents obtain information modeled explicitly, or are individuals simply assumed to know these variables?	Sensing is often assumed to be local, but can happen through networks or can even be assumed to be global.	Simply assumed to know them.
		**II.iv.e Are costs for cognition and costs for gathering information inclu-ded in the model?**		No.
		**II.v.a Which data does the agent use to predict future conditions?**	**Extrapolation from experience, from spatial observations**	Buyers assess their fishers' catch in the next time step by aggregating the catch of its fishing crew in the current time step.
		II.v.b What internal models are agents assumed to use to estimate future conditions or consequences of their decisions?		None.
		**II.v.c Might agents be erroneous in the prediction process, and how is it implemented?**	**(External) uncertainty, (internal) capability of the agent**	The assessed catch will be somewhat erroneous because of changes in stock and number of active fishers.
	II.vi Interaction	II.vi.a Are interactions among agents and entities assumed as direct or indirect?	Direct interactions, indirect interactions (mediated by the environment / **the market, auction)**	Direct between fishers in a co-op or in a PC relationship. Direct between market-buyer. Direct between fisher and biological entity. Indirect between buyer and fish-stock, indirect between fisher and market.
		**II.vi.b On what do the interactions depend?**	**Spatial distances (neighborhood), networks, type of agent**	On their network and type of agent.<br>If they are in a PC or co-op type of network.
		II.vi.c If the interactions involve communication, how are such communications	**Explicit messages (Matthews et al., 2007)**	The interactions involve communication but it is not explicitly represented.

		represented?		
		**II.vi.d If a coordination network exists, how does it affect the agent behaviour? Is the structure of the network imposed or emergent?**	Centralized vs. decentralized, group based tasks	The cooperative structure is imposed on fishers within a co-op and influences which other fishers they interact with (e.g. form loyalty, share economic returns). The PC structure is also imposed on six random inactive fishers, where the financially strongest inactive fisher becomes the fish buyer. The structure is hereafter emergent.
	II.vii Collectives	II.vii.a Do the individuals form or belong to aggregations that affects, and is affected by, the individuals? Does the modeller impose these aggregations or do they emerge during the simulation?	Social groups, human networks and organizations	A fisher can be a member of a co-op, which the modeler imposes (see above II.vi). Or a fisher can be a member of a PC, which the modeler imposes (see above II.vi).
		II.vii.b How are collectives represented?	Collective as emergent property vs. as a definition by the modeler (separate kind of entity with its own state variables and traits)	The modeler defines the collective, i.e. the co-op. The co-ops have links between fishers and are not separate entities per se. However they do have some state variable as a co-op budget that is effective only within the co-op. They also form loyalty to the co-op over time.
	**II.viii Heterogeneity**	**II.viii.a Are the agents heterogeneous? If yes, which state variables and/or processes differ between the agents?**	**Would an exchange of one agent with another at the beginning have an effect on the simulation?**	Fishers are heterogeneous. The variables are; fishing skills, reliability, and, if in a co-op, their initial loyalty. Buyers are homogenous. The processes between buyers and fishers are different.
		**II.viii.b Are the agents heterogeneous in their decision-making? If yes, which decision models or decision objects differ between the agents?**		They are homogenous.
	II.ix Stochasticity	II.ix.a What processes (including initialization) are modeled by assuming they are random or partly random?		The cheating process includes stochasticity. The selection process to form a PC or co-op includes randomly selected agents.<br>In the stochastic environment catchability is stochastic.

	II.x Observation	II.x.a What data are collected from the ABM for testing, understanding, and analyzing it, and how and when are they collected?		Primarily the number of fishers active in each organization is analyzed over the last thirty years of the simulation. Then capital, loyalty, reliability, fishing skills, why coops went out of business, number of PCs that went out of business, number of active PCs and c-ops, state of fish stock, time step, time steps alive for co-ops and PCs.<br><br>Every 100timestep was saved to a cvs file.
		II.x.b What key results, outputs or characteristics of the model are emerging from the individuals? (Emergence)		Ratio of PC versus coops; income distribution in community (fishers in co-ops capital, fishers in PC capital, buyers capital, coops capital), state of the fish population, loyalty in PC's and co-ops.
**III) Details**	**II.i Impl.**	**III.i.a How was the model implemented?**		Netlogo [8]
		**III.i.b Is the model accessible?**		Yes in OpenABM [9,10]
	III.ii Initialization	III.ii.a What is the initial state of the model world, i.e. at time t=0 of a simulation run?	Types and numbers of entities including the agents themselves, values / random distribution of their state variables	100 inactive fishers. Different distributions and means of reliability among fishers. Different means of initial loyalty for co-ops.
		III.ii.b Is initialization always the same, or is it allowed to vary among simulations?		We modify initial settings of reliability (mean and standard deviation), initial loyalty (mean), catchability (fluctuating versus stable), and of seasonality (stock changing per 6 months or stable).
		III.ii.c Are the initial values chosen arbitrarily or based on data?	References to data if any, **stakeholder choice**	Systematically to assess their impact on model outcomes
	III.iii Input Data	III.iii.a Does the model use input from external sources such as data files or other models to represent processes that change over time?	Observed time series e.g. annual rainfall, time series generated by other models,<br><br>not: parameter values, initial values of state variables	No.

		III.iv.a What, in detail, are the submodels that represent the processes listed in 'Process overview and scheduling'?	Equations, algorithms, additional information	Submodel: the fish population was represented by a logistic growth function, see S2 Calibrations.
		III.iv.b What are the model parameters, their dimensions and reference values?	Tables of parameters	See S2 Calibration for equations, calibration, functions, and parameter settings.
		III.iv.c How were submodels designed or chosen, and how were they parameterized and then tested?	Justifications, references to literature, independent implementation, testing, calibration, analysis of submodels	See S1 Table.

References S3 Table

1. Grimm V, Berger U, DeAngelis DL, Polhill JG, Giske J, Railsback SF. The ODD protocol: A review and first update. Ecol Model. 2010;221: 2760–2768. doi:16/j.ecolmodel.2010.08.019

2. Ostrom E. Governing the commons. Cambridge: Cambridge University Press; 1990.

3. Jensen MC, Meckling WH. Theory of the firm: Managerial behavior, agency costs and ownership structure. J Financ Econ. 1976;3: 305–360. doi:10.1016/0304-405X(76)90026-X

4. Clark CW. Mathematical bioeconomics: the optimal management of renewable resources. N Y. 1976;129.

5. Basurto X. Finding Order Amid Complex Small-Scale Fisheries Self-Governance Arrangements [Internet]. Rochester, NY: Social Science Research Network; 2016. Report No.: ID 2873306. Available: https://papers.ssrn.com/abstract=2873306

6. Bennett A. The influence of neoliberalization on the success and failure of fishing cooperatives in contemporary small-scale fishing communities: A case study from Yucatán, Mexico. Mar Policy. 2016;

7. Basurto X, Bennett A, Weaver AH, Rodriguez-Van Dyck S, Aceves-Bueno J-S. Cooperative and Noncooperative Strategies for Small-scale Fisheries' Self-governance in the Globalization Era: Implications for Conservation. Ecol Soc. 2013;18: 38.

8. Wilensky U. Netlogo [Internet]. Evanston, IL: Center for Connected Learning and Computer-Based Modeling, Northwestern University; 1999. Available: http://ccl.northwestern.edu/netlogo/

9. Lindkvist E, Basurto X, Schlüter M. SMILI: Small-scale fisheries Institutions and Local Interactions (Version 1). CoMSES Comput Model Libr. 2017; Available: https://www.openabm.org/model/5513/version/1/view

10. OpenABM. Open Agent Based Modeling Consortium | ... a node in the CoMSES Network [Internet]. [cited 13 Feb 2016]. Available: https://www.openabm.org/
